# Bobath Approach in Telemedicine Versus Conventional Treatment in Newborns Discharged from the NICU During the COVID-19 Pandemic

**DOI:** 10.1177/26924366251378097

**Published:** 2025-09-18

**Authors:** Lilia Martínez Molina, Solange Gabriela Koretzky, Guillermo Vargas López, María Luisa Peralta Pedrero, María de Lourdes Martín López, Juan Garduño Espinosa

**Affiliations:** ^1^ Medical, Dental, and Health Sciences Program, UNAM: Universidad Nacional Autonoma de Mexico Direccion General de Estudios de Posgrado, Mexico City, Mexico.; ^2^ Department of Clinical Research, Hospital Infantil de México Federico Gómez, Mexico City, Mexico.; ^3^ Department of Research, Hospital Dermatológico Ladislao de la Pascua, Mexico City, Mexico.; ^4^ Department of Rehabilitation, Hospital Infantil de México Federico Gómez, Mexico City, Mexico.

**Keywords:** Bobath approach, COVID-19, newborns, telemedicine

## Abstract

**Background:**

Prematurity is one of newborns’ leading causes of neurological damage. Although physical therapy reduces possible sequelae, there is insufficient scientific evidence on these treatments because they are delivered through telemedicine.

**Objective:**

To compare how newborns treated with the Bobath approach (BA) and those under conventional treatment scored at the end of 6 months of monitoring neurological warning signs and overall movement quality during the COVID-19 pandemic, given that all treatments were delivered through telemedicine.

**Materials and Methods:**

This analytical study involved 20 patients managed with the BA and 20 patients undergoing conventional treatment (*n* = 20). Three examinations and two treatment blocks were performed. The telemedicine sessions carried out through the Zoom platform were mainly focused on the relative training of hands-off techniques. Male and female newborns at high neurological risk were included.

**Results:**

Our findings show that both treatment arms delivered by telemedicine are effective (*p* = 0.6).

**Conclusion:**

Studies with larger sample sizes are necessary to investigate the impact of telemedicine on the economy and travel time.

## Introduction

From 2012 to 2018, a 2.3% increase in the survival rate of newborns born at less than 27 weeks of gestation was reported^1^ due to technological advancements in the newborn intensive care unit (NICU). Children at high neurological risk (HNR) are those more likely to present physical, cognitive, social, and personality impairments that may have short-term, medium-term, and long-term effects.^2^ Neurological damage occurs after a perinatal event and alters the structural and functional integrity of the newborn’s developing nervous system.^3,4^ Prematurity is the leading risk factor for neurological damage. The global rate of premature births in children born in 2020 ranges from 4% to 16%, and 200,000 premature births are reported in Mexico every year.^5,6^ Moreover, congenital disabilities are the second most frequent type of disability in Mexico, and it affects 27% of the Mexican population. This leads to a public health issue: according to data from Instituto Nacional deEstadística y Geografía (INEGI) (2017), 17.9% of subjects will develop some disability, 11.1% will present motor alterations, 6% will suffer from cerebral palsy, and 5.4% will show cognitive disabilities.^7^

Rehabilitation aims at reducing possible sequelae and optimizing maximum functional independence to become a productive member of society. Various physical therapy methods for newborns are used worldwide, but scientific evidence for these therapies is limited.^8^ The Bobath approach (BA) is a specialized therapy addressing motor and postural alterations derived from central nervous system (CNS) lesions. It focuses on inhibiting pathological reflexes, normalizing muscle tone, achieving regular coordination of voluntary movements, independent movement, and manual dexterity. As a result, patients have better functional abilities and an increased ability to move, making their functionality as normal as possible from a perceptual, cognitive, and emotional point of view. In addition, BA can prevent secondary disorders and deformities.^9–11^ However, the early stimulation program (ESP) is the conventional therapy used in our institution. ESP was initially used for children at socio-environmental risk in developed countries to have a purposeful life and be integrated into society. In the 1990s, it was first applied to children at biological risk, such as HNR patients.^12–14^ As a result, the concept of early intervention was introduced as a set of educational techniques oriented toward children and their families; its objective is to minimize the lack of stimuli and learning problems and to maximize the children’s psychophysical possibilities through a regulated, systematic, and continuous stimulation in all development areas without forcing the logical course of CNS maturation. The ESP is applied in the population from 0 to 3 years of age, and it consists of five phases to stimulate developmental milestones: phase 1 corresponds to neck control, phase 2 to trunk control, phase 3 to scooting, phase 4 to crawling, and phase 5 to initiation of standing and walking.^15,16^

The COVID-19 pandemic increased the use of telemedicine, allowing us to work with patients from different locations and continue with their rehabilitation in their social environment, mitigating the adverse effects of lockdown. These telehealth services can be provided in real time or asynchronously.^17,18^ In a systematic review published by Camden et al.^19^ mentions that telerehabilitation is a promising approach for rehabilitation services for the pediatric stage. Still, more research is required to explore the contexts, populations, and interventions that render telerehabilitation the most effective and cost-effective and also concludes that telerehabilitation might be particularly effective when a coaching approach is used. The primary strategy in telehealth is coaching. Positive psychology defines the concept of “health and wellness coaching” as the application of a methodology to “help patients get the knowledge, skills, tools, and confidence to become active participants in their care so that they can achieve their health goals.” This is considered a paradigm shift from traditional interventions.^20–22^ In pediatric physical therapy, coaching is used to strengthen the abilities of adult relatives to support children in their development within the context of daily activities and routines.^23–27^ Hsu et al.^28^ published that early intervention demonstrates that telehealth can improve provider fidelity to the role as coach, yield equivalent outcomes for social and behavioral outcomes, and deliver effective treatment for motor impairments. Subjective benefits cited by recipients of telehealth intervention include flexibility, accessibility, and versatility. For example, telehealth practitioners could facilitate visits during nontraditional times, such as critical daily routines like dressing, bathing, and mealtimes, to provide relevant situational coaching. Evidence supports that telehealth services are well received by the patients, do not increase caregiver burden, and may confer functional beneﬁts for certain patients^22^; however, interpretation of the available literature is limited by variation in methodology and approaches and in the evaluation of patient improvement. The present study compared the baseline score with the score after monitoring neurological warning signs and general movement quality for 6 months. HNR newborns discharged from the NICU treated with the BA and conventional treatment (ESP) were considered. All services were provided through telemedicine during the COVID-19 pandemic.

## Materials and Methods

An analytical study was conducted. It included examinations performed before and after 6 months of treatment and was approved by the hospital’s ethics committee under approval number HIM/2021-086SSA1793.

Recruitment was carried out in the NICU by direct invitation to the patient’s relatives, who were informed about the research and signed informed consent. Inclusion criteria: being an HNR newborn (hyperbilirubinemia, neonatal sepsis, respiratory distress syndrome, hypoglycemia, neonatal diabetes, intrauterine growth restriction, prematurity, dehydration, intestinal atresia, post-COVID); male and female patients; and living in Mexico City or abroad. Exclusion criteria: being a newborn with unstable cardiopathies, congenital malformations (clubfoot, myelomeningocele) or Down syndrome, lacking access to the internet or electronic devices for communication, and showing neurological warning signs in the baseline examination. Elimination criteria: consent withdrawal by a family member, hospitalization for more than 2 weeks, and protocol abandonment. It must be highlighted that patients were not excluded from the statistical analysis since it was performed on an intention-to-treat basis.

**FIG. 1.**
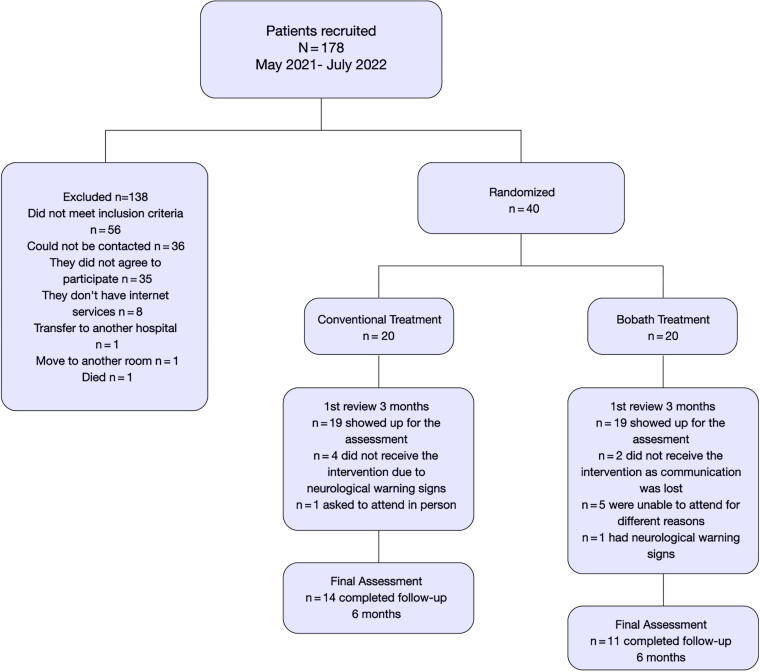
Flow diagram of participants.

The sample size was estimated using α = 95 and β = 20. The work by Blauw-Hospers (2011) was taken as a reference for considering signs of neurological damage as an outcome variable. A total of *n* = 40 was obtained for both groups: *n* = 20 was recruited for BA and *n* = 20 for ESP. An online software application on the random.org website randomly assigned a type of therapy. Three face-to-face examinations were performed: upon admission (baseline), after 3 months of treatment (intermediate), and after 6 months of monitoring (final). A 4-min video was recorded to analyze the patient’s overall quality of movement; a pediatric neurologist evaluated this recording. In addition, Hammersmith examinations were used to assess warning signs, and a qualified rehabilitation physician onducted these assessments. In the baseline examination, the Hammersmith neonatal examination was performed according to the following reference scores: 34 points (maximum), 24–33 points (optimal), 17–23 points (suboptimal), and 16 points or less (neurological warning signs). In the intermediate and final examinations, the Hammersmith examination was utilized with the following scores: 78 points (maximum), 57–77 points (optimal), 51–56 points (suboptimal), and 50 points or less (signs of neurological damage). In the assessment of overall movement, results were reported as normal movement, poor repertoire, absence of fidgety movements, chaotic movements, and synchronous spasms. Patients who scored 16 points or less in the baseline examination and exhibited synchronous spasms were not recruited, as a personalized follow-up was necessary. The same occurred with participants scoring 50 points or less and exhibiting poor repertoire movements in the intermediate examination; in this case, patients were directed to personalized follow-up. Patients were given a block of treatment—a 30-min telemedicine session once a week for 1 month and 2 months of work at home—after the baseline and the intermediate examinations. The telemedicine sessions were carried out via Zoom, and treatment was explained using a screen, focusing on training the family members to perform the treatment maneuvers. Movement quality was assessed by a neuropediatrician using a 4-min video, and the same was performed in both treatment arms. The Hammersmith assessment was conducted by a rehabilitation physician, lasting approximately 30–40 min, and performed similarly in both treatment arms. Treatment sessions were delivered by two different therapists, experts in both treatments; each therapist treated 20 patients corresponding to their treatment arms for 30 min. Figure 1 shows the flow diagram of the participants.

### Data analysis

Sociodemographic variables were analyzed using descriptive statistics; chi-squared test, Student’s *t*-test, and Mann–Whitney U test were used as appropriate for the differences between both groups. The level of statistical significance was established with *p* ≤ 0.05, and the analysis was performed with the SPSS Statistics 29 package.

## Results

A total of 20 patients were included in the BA arm, 100% of whom underwent the baseline examination. Of these patients, 19 (95%) completed the first block of treatment, and 1 (5%) did not stay in touch or complete the first block of sessions. The 19 patients mentioned above participated in the initial assessment for the second treatment block. Two patients (10%) did not stay in touch, five patients (25%) could not attend for different reasons, and one patient (5%) presented warning signs and left the study protocol. As a result, 11 patients (55%) underwent the second treatment block and completed the 6-month monitoring period.

In the conventional treatment arm (*n* = 20), 100% of patients underwent baseline examination and followed the first treatment block. One patient (5%) lost contact, and 19 (95%) returned for the initial evaluation. Of these 19 participants, 4 (20%) showed warning signs in the first assessment and left the protocol, while 1 (5%) preferred face-to-face treatment. As a result, 14 (70%) patients underwent the second treatment block and completed the 6-month monitoring period. The mean age was 50 days in the BA group and 43 days in the conventional treatment group. Regarding the diagnostic variable, the most frequent were gastrointestinal diseases, including intestinal atresia, anorectal malformation, sigmoidoscopy, gastroschisis, necrotizing enterocolitis, ileostomy, diaphragmatic hernia, gastroesophageal fistula, recto-vestibular fistula, and digestive tract bleeding in both groups.

As shown in Table 1, there were no significant differences in sociodemographic variables between the treatment arms. Regarding the variables related to parents, parents’ ages were observed to be similar in both treatment groups. The variable related to the mother’s educational level differs: Mothers in the conventional group achieved a higher academic level, whereas most mothers in the BA group achieved an elementary education level. However, this difference was not statistically significant (*p* = 0.3). The conventional treatment group lived in Mexico City, while the BA group lived in other parts of Mexico. Regarding socioeconomic level, the traditional treatment group was level 2, and the Bobath treatment group was level 1. Table 2 summarizes the results of the variables associated with the participants’ parents. No significant differences were found between the two treatment arms in the analysis of neurological warning signs (Table 3) or overall quality of movement.

**Table 1.** Participants’ Sociodemographic Variables

Variable	Conventional treatment (ESP) *n* = 20	Bobath approach *n* = 20	Sig.
Age (days), median (IQR)	43 (24)	50 (59)	0.20^a^
Diagnosis, no. (%)			
Digestive system diseases	12 (60%)	10 (50%)	0.58^b^
Other diseases	3 (15%)	4 (20%)	
Cardiovascular diseases	0 (0%)	2 (10%)	
Respiratory diseases	2 (10%)	3 (15%)	
CNS diseases	3 (15%)	1 (5%)	
Gestational weeks, median (IQR)	37 (3)	37 (6)	0.9^a^
Sex, no. (%)			
Male	10 (50%)	11 (55%)	0.1^c^
Gas levels at birth (pH), median (IQR)	7.2 (0.1)	7.3 (0.1)	0.09^a^
Apgar score at birth, median (IQR)	8.9 (1.1)	8.8 (2.1)	0.3^a^
Head circumference at birth, median (IQR)	33.5 (4)	33 (3)	0.4^a^
Days at NICU, median (IQR)	19 (20)	29 (49)	0.3^a^
Ventilation in hospital, no. (%)			
Yes	13 (65%)	12 (63.2%)	0.1^c^
No	7 (35%)	7 (36.8%)	
Ventilation time (days), median (IQR)	2 (4)	2 (8)	0.9^a^
Physical therapy at NICU, no. (%)			
Yes	14 (70%)	14 (70%)	0.1^c^
No	6 (30%)	5 (25%)	

^a^ Chi-squared test.

^b^ Fisher’s exact test.

^c^ Mann–Whitney U test.

CNS, central nervous system; ESP, early stimulation program; IQR, interquartile range; NICU, newborn intensive care unit; Sig., significance.

**Table 2.** Sociodemographic Variables of Participants’ Parents

Variable	Conventional treatment (ESP) *n* = 20	Bobath approach *n* = 20	Sig.
Mother’s age (years), mean (SD)	25 (8.3)	24 (7.0)	0.1^a^
Father’s age (years), mean (IQR)	26 (10)	26.5 (12)	0.8^b^
Mother’s educational level, no. (%)			
Elementary education	5 (25%)	8 (40%)	0.3^c^
Secondary education	6 (30%)	7 (35%)	
Higher education	9 (45%)	5 (25%)	
Father’s educational level, no. (%)			
Elementary education	6 (31.6%)	8 (42.1%)	0.7^c^
Secondary education	6 (31.6%)	6 (31.6%)	
Higher education	7 (36.8%)	5 (26.3 %)	
Place of residence, no. (%)			
Mexico City	8 (40%)	4 (20%)	0.3^c^
Mexico (country)	7 (35%)	10 (50%)	
Abroad	5 (25%)	6 (30%)	
Socioeconomic level, no. (%)			
Level 1	7 (35%)	10 (55.6%)	0.3^c^
Level 2	11 (55%)	6 (33.3%)	
Level 3	2 (10%)	2 (11.1%)	

^a^ Student’s *t-*test.

^b^ Mann–Whitney U test.

^c^ Chi-squared test.

ESP, early stimulation program; SD, standard deviation.

**Table 3.** Variables of Neurological Warning Signs

	Conventional treatment (ESP)	Bobath approach	Sig.
Hammersmith neonatal examination	Baseline examination, *n* = 20	Baseline examination, *n* = 20	
34 score = maximum			
24–33 score = optimal			0.6^a^
17–23 score = suboptimal	23.9	23.3	
Score of 16 or less = neurological warning signs			
Hammersmith infant examination	First assessment, *n* = 19	First assessment, *n* = 19	
78 score = maximum			
57–77 score = optimal	61.5	62.5	0.5
51–56 score = suboptimal			
Score of 50 or less = neurological warning signs			
Hammersmith infant examination	Second assessment, *n* = 14	Second assessment, *n* = 11	
57–77 score = optimal	71.4	72.2	0.6^a^
51–56 score = suboptimal			
Score lower than 50 = neurological warning signs			

^a^ Student’s *t*-test.

ESP, early stimulation program.

## Discussion

The COVID-19 pandemic made it necessary to explore telemedicine’s results in different health care areas.^18,20^ In pediatric neurological rehabilitation, survey studies on the use of telemedicine before and after COVID-19 are reported in terms of user satisfaction; however, no specific techniques or results are analyzed since direct manipulation is believed to be the best way to help pediatric patients,^21,22^ another study reported that before COVID-19, the percentage of pediatric physiatrist employing telehealth was quite similar to pediatricians and pediatric subspecialists at approximately 15%. This number rose drastically to almost 100% in the following months.^22^ The objective of our study was to compare the results obtained from the BA and the conventional treatment in 40 premature newborns. The main finding is that there were no significant differences between the two treatments, suggesting that telemedicine shows positive results when provided by a qualified physiotherapist using a coaching tool and a hands-off approach and collaborating with the primary caregiver through close follow-up. This leads to a reduction in neurological signs, a shorter travel time (which may exceed 2 h in Mexico City), and a decrease in the economic costs associated with transport from different parts of the country. A previous study^3^ reported a higher percentage of mothers with secondary educational levels. However, in our study, many mothers achieved a higher academic level.

Regarding the variable of hospitalization days, it was higher in the BA group than in the conventional treatment group. However, this was not statistically significant; therefore, comorbidities associated with their condition may have impacted outcome variables. One of the strengths of our study lies in the fact that we closely monitored adherence to the procedural protocol in both treatment arms. In addition, different strategies have been used to achieve an adequate methodology and adherence to telemedicine treatment, as suggested by Galea in her research about telemedicine in rehabilitation.^21^ Another strength of our research is that telemedicine made it possible to incorporate the coaching tool and the hands-off technique, empowering relatives with different strategies to naturally optimize patients’ evolutionary neurodevelopment, as stated by Akhbari Ziegler in the Program COPing with and CAring (COPCA). In this case, the main challenge is the self-production of motor development and its implementation in the patient’s daily life with minimal manipulation from the physiotherapist.^23,27^

A limitation of our study was the sample size and the number of lost participants; therefore, studies with a larger sample size should be conducted to obtain results supporting both methods. However, it is worth mentioning that early intervention is critical, a public health issue leading to different developmental deviations that can be reduced if the intervention starts from birth.

Our results show that both treatments help to decrease neurological signs and enhance the neurological development of newborns. This innovative research includes telemedicine and incorporates the coaching tool and hands-off techniques during the COVID-19 pandemic. In addition, it achieves the objective of providing evidence-based and cutting-edge physiotherapy.

Further studies with a larger sample size should be conducted to reduce sequelae in newborns with HRN. Congenital disability is a public health issue both in Mexico and worldwide. Therefore, it is crucial to have more information on the use of telemedicine and its impact on the economy, travel time, and the improvement and monitoring of patients.

## Data Availability

The data that support the findings of this study are available upon request from the corresponding author.

## Abbreviations Used

BA: The Bobath approach
CNS: Central nervous system
ESP: Early stimulation program
HNR: High neurological risk
NICU: Newborn intensive care unit
